# First-line oxaliplatin-based chemotherapy and nivolumab for metastatic microsatellite-stable colorectal cancer—the randomised METIMMOX trial

**DOI:** 10.1038/s41416-024-02696-6

**Published:** 2024-04-25

**Authors:** Anne Hansen Ree, Jūratė Šaltytė Benth, Hanne M. Hamre, Christian Kersten, Eva Hofsli, Marianne G. Guren, Halfdan Sorbye, Christin Johansen, Anne Negård, Tonje Bjørnetrø, Hilde L. Nilsen, Jens P. Berg, Kjersti Flatmark, Sebastian Meltzer

**Affiliations:** 1 Department of Oncology, Akershus University Hospital, Lørenskog, Norway; 2 Institute of Clinical Medicine, University of Oslo, Oslo, Norway; 3 Health Services Research Unit, Akershus University Hospital, Lørenskog, Norway; 4 Department of Research, Sørlandet Hospital, Kristiansand, Norway; 5 Department of Oncology, St Olav’s Hospital, Trondheim, Norway; 6 Department of Clinical and Molecular Medicine, Norwegian University of Science and Technology, Trondheim, Norway; 7 Department of Oncology, Oslo University Hospital, Oslo, Norway; 8 Department of Oncology, Haukeland University Hospital, Bergen, Norway; 9 Department of Clinical Science, University of Bergen, Bergen, Norway; 10 Department of Radiology, Akershus University Hospital, Lørenskog, Norway; 11 Department of Clinical Molecular Biology, Akershus University Hospital, Lørenskog, Norway; 12 Department of Medical Biochemistry, Oslo University Hospital, Oslo, Norway; 13 Department of Gastroenterological Surgery, Oslo University Hospital, Oslo, Norway; 14 Department of Tumour Biology, Oslo University Hospital, Oslo, Norway

**Keywords:** Colorectal cancer, Cancer immunotherapy

## Abstract

**Background:**

We evaluated first-line treatment of metastatic microsatellite-stable colorectal cancer with short-course oxaliplatin-based chemotherapy alternating with immune checkpoint blockade.

**Methods:**

Patients were randomly assigned to chemotherapy (the FLOX regimen; control group) or alternating two cycles each of FLOX and nivolumab (experimental group). Radiographic response assessment was done every eight weeks with progression-free survival (PFS) as the primary endpoint. Cox proportional-hazards regression models estimated associations between PFS and relevant variables. A *post hoc* analysis explored C-reactive protein as signal of responsiveness to immune checkpoint blockade.

**Results:**

Eighty patients were randomised and 38 in each group received treatment. PFS was comparable—control group: median 9.2 months (95% confidence interval (CI), 6.3–12.7); experimental group: median 9.2 months (95% CI, 4.5–15.0). The adjusted Cox model revealed that experimental-group subjects aged ≥60 had significantly lowered progression risk (*p* = 0.021) with hazard ratio 0.17 (95% CI, 0.04–0.76). Experimental-group patients with C-reactive protein <5.0 mg/L when starting nivolumab (*n* = 17) reached median PFS 15.8 months (95% CI, 7.8–23.7). One-sixth of experimental-group cases (all *KRAS/BRAF*-mutant) achieved complete response.

**Conclusions:**

The investigational regimen did not improve the primary outcome for the intention-to-treat population but might benefit small subgroups of patients with previously untreated, metastatic microsatellite-stable colorectal cancer.

**Trial registration:**

ClinicalTrials.gov number, NCT03388190 (02/01/2018).

## Background

Owing to an ageing population, colorectal cancer (CRC) is a common malignancy with a sharp rise in incidence from the age of 60 [1]. Immune checkpoint blockade (ICB) is efficacious in the small CRC subgroup of patients with highly immunogenic disease, the microsatellite-instable/mismatch repair (MMR)-deficient entity [2, 3]. Also a rare patient subgroup with mutations in polymerase ε (*POLE*) or δ1 (*POLD1*), associated with a hypermutated phenotype and mostly observed in microsatellite-stable (MSS)/MMR-proficient tumours [4], shows ICB responsiveness [5]. ICB is, however, considered inefficacious for the majority of patients presenting MSS/MMR-proficient CRC, which causes low tumour antigenicity [6] and unlike the majority of metastatic microsatellite-instable/MMR-deficient CRC cases [2], often co-exists with high *RAS/BRAF*-driven oncogenic activity [7, 8]. Unresectable abdominal metastases commonly reflect a severe disease course [9]. A retrospective analysis of patients with unresectable metastatic MSS-CRC given ICB indicated that the presence of liver metastases was the most significant variable associated with rapid disease progression [10]. ICB responsiveness in MSS-CRC is considered more likely for lung metastases than liver metastases [11, 12].

Our previous findings for initial 2–4 cycles of oxaliplatin-containing chemotherapy in locally advanced or early metastatic CRC support a notion that oxaliplatin may invoke tumour-defeating immunity [13, 14]. Specifically, patients who presented unresectable single-organ liver metastases as the first metastatic event, given oxaliplatin as hepatic arterial infusion chemotherapy and responding with a rapid rise in a circulating anti-tumour immune factor, were alive 8-12 years later [14].

In the METIMMOX trial, patients with previously untreated, unresectable abdominal metastases from MSS-CRC were randomly assigned to short-course oxaliplatin-based chemotherapy (the Nordic FLOX regimen) alternating with ICB (nivolumab) or standard FLOX chemotherapy. Here we report the main efficacy and safety outcomes.

## Methods

### Study design and participants

The METIMMOX trial (Colorectal Cancer METastasis – Shaping Anti-Tumour IMMunity by OXaliplatin) was an investigator-initiated, open-label, randomised phase 2 trial, approved and conducted as per Norwegian legislation (Supplementary methods). Patients, with no upper age limit to recruit subjects reflecting population-based incidence rates, had previously untreated, unresectable metastatic colorectal MSS adenocarcinoma and were enroled at five hospitals. Essential study inclusion criteria were age ≥18 years, measurable infradiaphragmatic (liver, peritoneal and/or nodal) metastatic manifestation(s) according to Response Evaluation Criteria in Solid Tumours version 1.1 (RECIST 1.1), and Eastern Cooperative Oncology Group performance status 0-1. In addition, C-reactive protein (CRP) <60 mg/L was required at study entry based on the observation that baseline CRP values above, as a pragmatic cutoff, had been found strongly associated with impaired prognosis in metastatic CRC [15]. A period <6 months since discontinuation of neoadjuvant or adjuvant oxaliplatin-containing chemotherapy and a history of autoimmune disease were main exclusion criteria. The complete list of inclusion and exclusion criteria can be found with the clinical trial registration (ClinicalTrials.gov Identifier: NCT03388190) and in the trial protocol, available from the corresponding author upon request.

### Procedures

The patients were block-randomised into the treatment arms with ratio 1:1 (Supplementary methods) with regard to primary tumour sidedness (right or left/rectum) and *RAS/BRAF* mutational status (wildtype or any mutation, determined according to clinically routine procedures in accredited molecular pathology laboratories). These procedures and other molecular procedures (testing of tumour MMR proteins and MSS status, and sequencing with the TruSight Oncology 500 DNA/RNA Assay for the assessment of tumour mutational burden (TMB) and *POLE*/*POLD1* mutations) are detailed in Supplementary methods. The METIMMOX trial schedule (Supplementary Fig. S1) was designed to reflect the prevailing clinical practice [16] and national guidelines for first-line therapy in metastatic CRC. Thus, the patients were assigned to eight cycles of the FLOX regimen Q2W (oxaliplatin 85 mg/m^2^ day 1 and bolus 5-fluorouracil 500 mg/m^2^ and folinic acid 100 mg days 1–2; control arm) or two cycles of FLOX Q2W before two cycles of nivolumab (240 mg flat dose) Q2W in an alternating schedule to a total of eight cycles (experimental arm). The ICB was administered without concomitant chemotherapy that might compromise an invoked anti-tumour immunity, resulting in only four cycles each of chemotherapy and nivolumab within a treatment sequence. For both trial arms, an active treatment sequence was followed by a break until disease progression and reintroduction of a new treatment sequence. The go-and-stop schedule (alternating active therapy and treatment breaks) was continued until the first confirmed disease progression on active therapy (progressive disease (PD)), an intolerable adverse event, consent withdrawal or death, whichever occurred first. Prespecified adverse events, according to the Common Terminology Criteria for Adverse Events version 4.0, entailed treatment modifications detailed in the protocol. An independent safety monitoring committee periodically reviewed the safety data. Tumour assessments were based on blinded independent central review according to RECIST 1.1 as the primary method and the consensus guidelines for assessment of response to immune-modulating therapies (iRECIST) as the subsidiary method, by means of CT scans repeated every 8 weeks throughout the study participation.

### Outcomes

The primary endpoint was progression-free survival (PFS), defined as the time from commencing the first FLOX cycle to the first documentation of PD (according to RECIST/iRECIST) on active therapy, determining failure of treatment strategy, or death from any cause, whichever occurred first. Data for patients who had not experienced PD on active therapy or not undergone metastasis surgery with curative intent were censored as of the date of the last imaging assessment, provided that study treatment was not recommenced following the surgery. Patients with relapse of metastatic disease and recommencing study treatment after metastasis surgery were followed until they reached a prespecified endpoint. The prespecified secondary endpoints were the objective response rate (ORR; the percentage of patients who achieved partial response (PR) or complete response (CR) according to RECIST/iRECIST) and duration of response (DOR) as recommended for ICB therapies [17] and defined by the interval from response initiation (when either PR or CR was first determined) to PD on active therapy. Safety (the incidence of grade 3-5 adverse events and grade 2 immune-related hepatotoxicity) and overall survival were also secondary endpoints. During the trial conduct we observed that CRP levels might decline over the initial treatment, encouraging a *post hoc* analysis of CRP as a signal of activity or failure of the investigational ICB schedule.

### Statistical analysis

Determination of sample size was performed based on PFS data from ICB studies in metastatic cancer available at the time of preparation of the protocol (September–December 2017), specifically in previously untreated patients with advanced non-small-cell lung cancer [18]. Extrapolating to first-line treatment of metastatic MSS-CRC, the primary efficacy hypothesis was that the experimental-arm treatment would lead to median PFS twice as long (18 months) compared to the median of approximately 9 months for historical control-arm treatments [16, 19]. Assuming the exponential distribution of survival functions, the median PFS estimates were converted to hazard ratio of 0.5. Allowing for 10% censoring rate of subjects, the required sample size was estimated to be 40 patients in each arm with 1:1 randomisation. Provided that the risk of progression in the experimental arm was 50% lower than in the control arm, this sample size was sufficient to show with the power of 80% it was significantly different from 1 at a significance level of 5% according to two-sided log-rank test. Further details on the statistical plan are given in Supplementary methods.

The prespecified efficacy and safety analyses were done on the protocol-defined intention-to-treat sample. As the primary analysis, PFS times were presented by Kaplan–Meier curves and median PFS times were compared between the study arms by log-rank test. Prespecified Cox proportional hazards regression models on the intention-to-treat sample were estimated to determine associations between PFS and relevant patient variables, as stratified by study arm, and reduced for excessive interactions by the Bayesian information criterion. Because the first two therapy cycles were identical in the control and experimental study arms (halfway towards the first radiographic reassessment), the per-protocol population included all subjects who adhered to treatment until the first reassessment to enable objective comparison of the regimens. The ORR and safety data were compared by the χ^2^-test (or Fisher’s exact test), the DOR and overall survival data using the log-rank test, the TMB data by the Mann–Whitney U test and the CRP data by the Kruskal–Wallis test. All tests were two-sided. The analyses were performed using STATA SE version 17 and GraphPad Prism version 9.5.1.

## Results

### Patients and treatment

The 80 patients were enroled between 29 May 2018 and 22 October 2021 (CONSORT diagram with details: Supplementary Fig. S2). Excluding ineligible patients who had been mistakenly randomised or did not receive any study intervention [20], 76 intention-to-treat subjects were randomly allocated between the study arms (thus, also comprising the safety population) with baseline characteristics given by Table 1 (individual tumour mutations in Supplementary Table S1). The primary objective—to demonstrate median PFS twice as long in experimental-arm patients compared to the control-arm patients—was not met.

**Table 1:** Patients’ baseline characteristics.

		All patients (*n* = 76)	Control-arm patients (*n* = 38)	Experimental-arm patients (*n* = 38)
		*n* (%)	*n* (%)	*n* (%)
Median age, years (minimum; maximum)		64.5 (38; 80)	65.0 (38; 79)	60.5 (43; 80)
Sex	Female	35 (46)	15 (39)	20 (53)
	Male	41 (54)	23 (61)	18 (47)
ECOG performance status	0	44 (58)	21 (55)	23 (61)
	1	32 (42)	17 (45)	15 (39)
Primary tumour sidedness	Right	22 (29)	11 (29)	11 (29)
	Left or rectum	54 (71)	27 (71)	27 (71)
*RAS/BRAF* status	Wildtype	21 (28)	9 (24)	12 (32)
	Mutant	55 (72)	29 (76)	26 (68)
Number of metastatic sites	1–2	46 (61)	22 (58)	24 (63)
	>2	30 (39)	16 (42)	14 (37)
Involved liver	No	13 (17)	6 (16)	7 (18)
	Yes	63 (83)	32 (84)	31 (82)

Patient characteristics of the treatment arms were balanced according to primary tumour site with 71% left-sided/rectal cases. In contrast, distribution of the other stratification parameter, the global *RAS/BRAF* mutational status (wildtype or any mutation, detailed in Supplementary Table S1) in the study arms was unbalanced, resulting from three cases incorrectly registered for mutational status at the computer-based allocation but corrected in the data analysis. The experimental arm was further characterised by more females and lower median age. *ECOG* Eastern Cooperative Oncology Group.

### The primary endpoint PFS

**Fig. 1:**
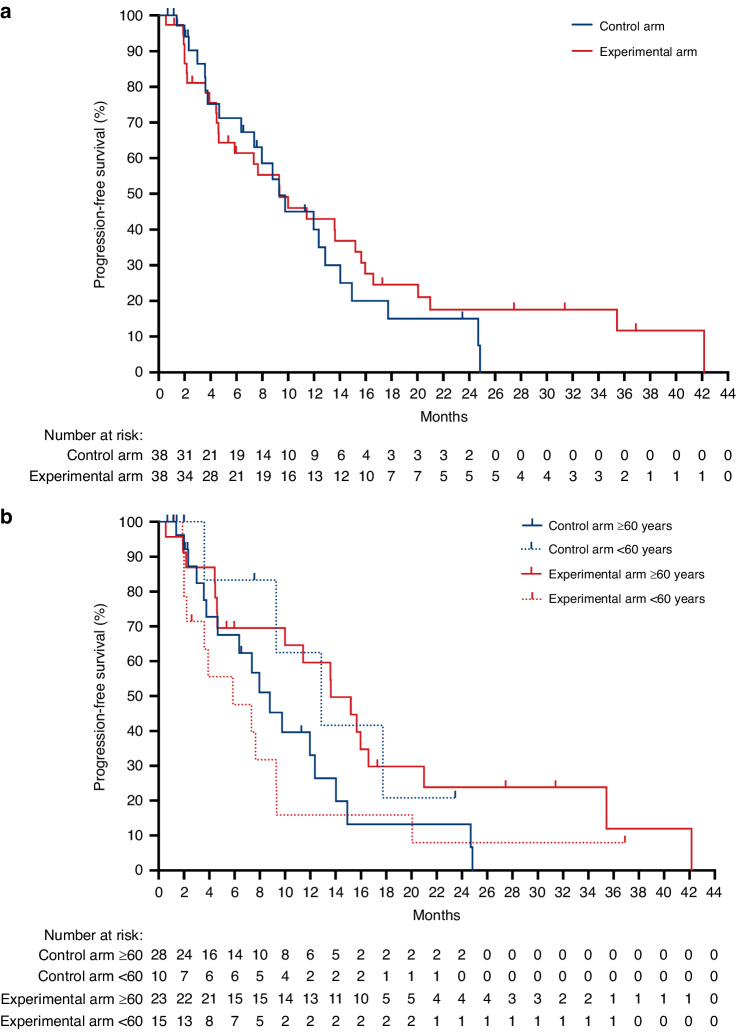
Kaplan–Meier curves of progression-free survival for the intention-to-treat population. The 76 cases were stratified by (**a**) study arm or (**b**) study arm and age; <60 years: *p* = 0.14, ≥60 years: *p* = 0.052 (log-rank test).

At the data cutoff on 30 October 2023, the study arms showed comparable PFS (*p* = 0.52; Fig. 1a)—control arm (*n* = 38): median 9.2 months (95% confidence interval (CI), 6.3-12.7); experimental arm (*n* = 38): median 9.2 months (95% CI, 4.5–15.0). No strong deviations from the proportional hazards assumption were identified. According to the adjusted Cox model (Table 2), the only significant interaction was between patient age (dichotomised to 60 years and older or younger than 60 years, typically used for this patient population) and treatment arm, where patients ≥60 years given alternating FLOX and nivolumab had lowered risk of progression with derived hazard ratio 0.17 (95% CI, 0.04–0.76), *p* = 0.021 (Supplementary Table S2: the individual hazard ratios for this interaction, Fig. 1b: the descriptive PFS curves). Reflecting infradiaphragmatic metastases as eligibility criterion, as much as 83% of the intention-to-treat population presented with involved liver (Table 1) and as separate patient variable with significantly increased risk of progression for experimental-arm subjects (*p* = 0.031, Table 2; Supplementary Fig. S3: the non-significant interactions by patient variables).

**Table 2:** Cox proportional hazards regression models for progression-free survival.

	Ctr. arm, Events/*n* (%)	Exp. arm, Events/*n* (%)	Unadjusted models		Adjusted model	
			Hazard ratio (95% CI)	*p*	Hazard ratio (95% CI)	*p*
Study arm						
Control (ref.)			0		0	
Experimental			0.81 (0.58)^a^	0.17	1.41 (0.64)^a^	0.029
Age, years						
<60 (38–59; ref.)	3/10 (30)	11/15 (73)	0		0	
≥60 (60–80)	17/28 (61)	17/23 (74)	0.63 (0.56)^a^	0.25	1.11 (0.67)^a^	0.096
Study arm × Age			–1.40 (0.68)^a^	0.039	–1.77 (0.77)^a^	0.021
Sex						
Female (ref.)	5/15 (33)	13/20 (65)	1		1	
Male	15/23 (65)	15/18 (83)	1.41 (0.80–2.49)	0.24	1.03 (0.52–2.06)	0.93
ECOG performance status						
0 (ref.)	10/21 (48)	17/23 (74)	1		1	
1	12/17 (71)	13/15 (87)	1.58 (0.91–2.76)	0.11	2.08 (1.09–3.96)	0.026
Primary tumour sidedness						
Right (ref.)	5/11 (45)	6/11 (55)	1		1	
Left or rectum	15/27 (56)	22/27 (81)	2.25 (1.07–4.74)	0.032	2.07 (0.88–4.86)	0.097
*RAS/BRAF* status						
Wildtype (ref.)	4/9 (44)	8/12 (67)	1		1	
Mutant	16/29 (55)	20/26 (77)	1.04 (0.56–1.93)	0.90	0.85 (0.41–1.80)	0.68
Number of metastatic sites						
1–2 (ref.)	10/22 (45)	15/24 (63)	1		1	
>2	10/16 (63)	13/14 (93)	2.02 (1.14–3.57)	0.016	2.25 (1.11–4.52)	0.0024
Involved liver						
No (ref.)	1/6 (17)	4/7 (57)	1		1	
Yes	21/32 (66)	26/31 (84)	3.99 (1.39–11.4)	0.010	3.65 (1.12–11.8)	0.031

*CI* confidence interval, *Ctr*. control, *ECOG* Eastern Cooperative Oncology Group, *Exp*. experimental, *ref*. reference.

^a^Regression coefficient (standard error).

### Tumour responses

**Fig. 2:**
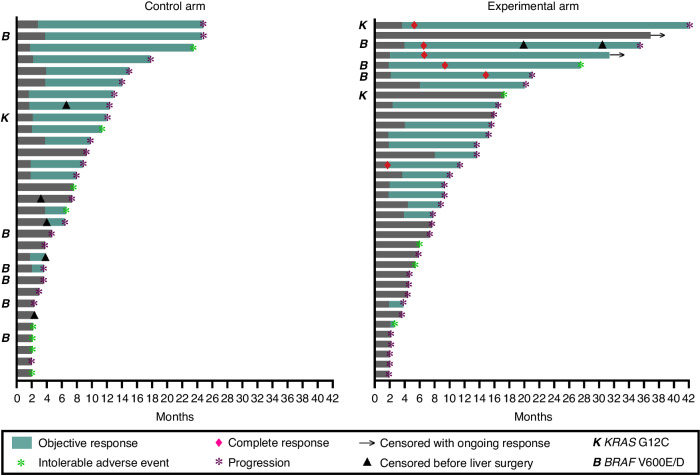
Duration of study participation and efficacy assessment for the per-protocol population of 67 cases. Objective response: Patient achieved partial or complete response according to the Response Evaluation Criteria in Solid Tumours version 1.1 (RECIST 1.1) and the consensus guidelines for assessment of response to immune-modulating therapies (iRECIST).

Secondary endpoints reflected tumour response patterns distinctive for chemotherapy only (tumour shrinkage caused by the cytotoxic mode of action) or the combined-modality treatment (tumour responses translating into various radiologic measures). Despite disparate ICB response patterns might pertain [21], the experimental-arm ORR of 47% (17 of 36 per-protocol cases) did not statistically differ (*p* = 0.16) from the control-arm ORR of 65% (20 of 31 per-protocol cases; Supplementary Fig. S4, Supplementary Table S3: by patient variables). With regard to the duration of study participation for the per-protocol cases (Fig. 2), the interval until either CR or PR was first determined was similar (*p* = 0.16)—control arm (*n* = 20): median 2.1 months (95% CI, 1.8–3.7); experimental arm (*n* = 17): median 2.1 months (95% CI, 1.8–3.9). Longer DOR (*p* = 0.045) was observed in the experimental arm with median 15.0 months (95% CI, 7.0–18.0) than in the control arm with median 9.0 months (95% CI, 2.0–11.0).

Of note, six experimental-arm patients (15.8%) had CR. As none of the control-arm patients achieved this outcome, the difference between the trial arms was significant (*p* = 0.027). The tumour MSS status for the experimental-arm CR cases was verified using complementary assays; five were females ≥60 years with right-sided primary tumour and all six were *RAS/BRAF*-mutant cases. Tumour sidedness was the only clinical characteristic significantly different from experimental-arm non-CR cases (*p* = 0.0035). None of the CR cases had tumour *POLE* or *POLD1* mutations (Table 3). The experimental-arm patient with longest PFS (41.6 months) carried *KRAS* G12C mutation (Fig. 2) and TMB of 9.4 mutations per megabase; the two other *KRAS*-mutant CR cases were TMB 9.4–10.9 (Table 3), intermediate between low and high TMB [22, 23]. Thirteen METIMMOX patients had tumour with *BRAF* V600E/D mutation, of whom ten were randomly allocated to the control arm (Supplementary Table S1) and had median PFS 3.7 months (95% CI, 3.0–7.3). All of three experimental-arm *BRAF*-mutant cases (TMB 6.2–11.8; Table 3) experienced CR with PFS 20.7–35.0 months (Fig. 2). As such, TMB (unknown for two subjects) was not different (*p* = 0.88) between the experimental-arm patients with (median, 8.0; minimum, 0.8; maximum, 12.2; *n* = 16) and without (median, 7.5; minimum, 0.8; maximum, 12.0; *n* = 18) objective tumour response.

**Table 3:** Tumour status for the experimental-arm cases with radiologic complete response.

Case – Sex, age (years)	Sidedness, mutation	PCR –OncoMate™ MSI Dx	PCR – Idylla™ MSI Test	IHC	NGS – TSO™ 500 Assay		
					MS status	TMB (mutations/ megabase)	*POLE*/ *POLD1*
Female, 60	Right, *KRAS* G12C	MSS	MSS	n.d.	MSS	9.4	wt
Female, 68	Right, *KRAS* G12V	n.d.	MSS	MMR-proficient	MSS	10.9	wt
Male, 71^a^	Left, *KRAS* G13D	MSS	MSS	n.d.	MSS	9.4	wt
Female, 70	Right, *BRAF* V600E/D	n.d.	MSS	MMR-proficient	MSS	6.2	wt
Female, 72	Right, *BRAF* V600E/D	MSS	MSS	n.d.	MSS	11.8	wt
Female, 73	Right, *BRAF* V600E/D	n.d.	MSS	n.d.	MSS	6.2	wt

*ICH* immunohistochemistry, *MMR* mismatch repair, *MS* microsatellite, *MSS* microsatellite-stable, *n.d*. not done, *NGS* next-generation sequencing, *TMB* tumour mutational burden, *wt* wildtype.

^a^Diagnosed with brain metastases 9.6 months after extracranial complete response was achieved and maintained.

### Safety and overall survival

As detailed in Supplementary Table S4, the percentage of patients reporting grade 3-4 adverse events during the chemotherapy cycles was comparable in the treatment arms. Of note in the experimental-arm population, 8% reported grade 3 diarrhoea and 18% grade 3 venous thromboembolism, compared to 3% and 11%, respectively, in the control-arm population (but not statistically different between the arms: *p* = 0.61 for the diarrhoea and *p* = 0.52 for the thromboembolism). Other grade 3 immune-mediated events occurred in 35% (13 of 37) of patients receiving nivolumab, but no grade ≥4 event was recorded.

Overall survival did not differ between the trial arms (*p* = 0.68; Supplementary Fig. S5, Supplementary Fig. S3: by patient variables)—control arm: median 14.6 months (95% CI, 10.6-23.2); experimental arm: median 20.7 months (95% CI, 15.9-24.9).

### Predictive value of CRP for PFS

This *post hoc* analysis was enabled by the recording of CRP values at each study visit for all participants. All had CRP <60 mg/L (maximum, 50.9) at study entry, as per protocol, but it had increased above 60 in five patients at start of therapy. The CRP measures for the intention-to-treat population declined over the initial FLOX treatment (*p* = 0.034; Supplementary Fig. S6). Experimental-arm patients with CRP within the reference limit (<5.0 mg/L) when starting nivolumab (*n* = 17) reached median PFS 15.8 months (95% CI, 7.8–23.7). The implications of the CRP dynamics with regard to PFS in control-arm and experimental-arm subjects (Supplementary Fig. S7) are further detailed in Supplementary results. Likewise, the dynamics of neutrophil counts, which might be interdependent, is described in Supplementary results.

## Discussion

The median PFS of 9.2 months for the METIMMOX experimental-arm subjects was no better than in the control group, failing the trial’s primary aim. This PFS is in line with historical data for the Nordic FLOX regimen [16] and irinotecan-based chemotherapy of a randomised trial’s control arm [24] in the first-line setting but clearly inferior to the median PFS of 11–12 months for the more intensified and toxic FOLFOXIRI regimen containing both oxaliplatin and irinotecan [22, 24]. None of these trials selected subjects for infradiaphragmatic disease manifestations, which unlike the METIMMOX study may have confounded study populations with cases presenting indolent lung metastases only.

The AtezoTRIBE trial was the first prospective study that randomised patients with metastatic MMR-proficient CRC to ICB together with first-line chemotherapy, which in this case was FOLFOXIRI and the angiogenesis inhibitor bevacizumab [22]. Atezolizumab was added to this combination in each of eight initial cycles before maintenance therapy without oxaliplatin and irinotecan; however, 6–7% of subjects had MMR-deficient CRC which may have accounted for the significantly improved PFS in the experimental group. The MMR-proficient cohort reached median PFS 12.9 months when given atezolizumab, which was 1.5 months improved from the treatment without [22]. The median overall survival of 30.8 months for the MMR-proficient cases given atezolizumab was not statistically superior to the control-arm outcome of median 26.9 months [25] but as much as 10 months longer than overall survival of the experimental-arm METIMMOX patients. The shorter overall survival for the METIMMOX patients was likely caused by lacking efficacy of the experimental regimen for certain patient subpopulations. Additionally, the median overall survival of only 14.6 months in the control arm strongly indicated some patient subgroups had received insufficient therapy.

Similarly, the CheckMate-9×8 trial randomised patients with metastatic CRC to first-line treatment (without breaks) with oxaliplatin-based chemotherapy and bevacizumab with or without nivolumab, with median PFS of 11.9 months—the percentage of MMR-deficient cases has not been disclosed [26]. Three single-arm trials have reported the addition of ICB to first-line standard therapy in metastatic MSS-CRC, with median PFS 11.1 months (*RAS/BRAF*-wildtype cases) [27], 9.8 months (*RAS/BRAF*-mutant cases) [28] and 8.2 months (*RAS*-mutant cases) [29]. In the last-mentioned trial, TMB above 5.8 was associated with longer PFS [29]. The AtezoTRIBE trial found that MMR-proficient cases with TMB ≥10 (5.6%) significantly benefitted from the addition of ICB [22].

It is said to be a consistent phenomenon across studies that ICB responsiveness in MSS-CRC is more likely for lung metastases than liver metastases [12, 30]. Different from the first-line trials adding ICB onto chemotherapy and seemingly also including patients with only lung metastases [22, 26–29], all METIMMOX patients presented infradiaphragmatic metastases and were in the experimental arm given alternating short-course chemotherapy and ICB in a total of four cycles each over approximately 4 months before treatment break. The break was imposed on account of control-arm FLOX tolerability by clinical experience and historical practice and might imply an insufficient number of chemotherapy cycles, particularly for the experimental-arm subjects. A meta-analysis of multiple randomised trials for advanced CRC indicated no detriment in survival for patients receiving intermittent treatment compared to continuous chemotherapy [31]. The METIMMOX go-and-stop schedule with de-intensified chemotherapy within a treatment sequence might even have been the essential benefit for patients with average-onset (age ≥60 years) disease by higher tolerance and so the longer DOR and significantly lowered progression risk. This may be of note also for other cancer populations predominated by elderly individuals. The oldest METIMMOX patient was 80; by comparison, the oldest AtezoTRIBE patient was only 67 and subjects ≥60 years with MMR-proficient disease had no benefit of the atezolizumab addition onto FOLFOXIRI and bevacizumab (nor had those aged <60) [22].

Cancer-induced systemic inflammation can be a dominant attribute of advanced CRC [32], conferring poor outcome in general [33] and impairing ICB efficacy in metastatic MMR-deficient disease [34]. By following the METIMMOX patients’ CRP measures in a *post hoc* analysis, it became evident that the initial two FLOX cycles could quench systemic inflammation and moreover, patients with CRP levels within the reference limit at the time of the first nivolumab administration seemed to have ICB-responsive disease. The impact in patients with metastatic MSS-CRC might entail the opportunity of ICB therapy based on CRP as a dynamic measure [35] during oxaliplatin-based chemotherapy, starting when this pragmatic inflammation marker has become sufficiently low.

The majority of the METIMMOX participants had left-sided/rectal primary tumour with *RAS*-driven oncogenic activity. However, five (all female) of the six experimental-arm CR cases had right-sided primary, among whom all *BRAF* V600E/D-mutant subjects (≥70 years). Primary *BRAF*-mutant tumours show high infiltration of cytotoxic T-cells, even for MSS-CRC [8]. Still, this tumour mutation is commonly a poor prognostic factor in metastatic CRC with median overall survival of approximately 1 year [36, 37]. Therapies directly targeting the intrinsically active tumour signalling pathways have resulted in median PFS of 5 months or shorter [38, 39]. A proof-of-concept study adding ICB to targeted therapies led to 25% ORR in MSS-CRC cases [40]. In this context, CR with PFS 20-35 months on a well-tolerated regimen consisting of de-intensified oxaliplatin-based chemotherapy and ICB repeatedly is notable, albeit only three patients provided the data. A number of ongoing trials evaluate combinations of RAF inhibitors with other molecularly targeted agents, some with the addition of oxaliplatin- or irinotecan-based chemotherapy or ICB [41].

Weaknesses of the METIMMOX study include the unblinded design for the clinical investigators, which was chosen to secure patient surveillance with regard to adverse events; for example, chemotherapy-induced colitis (requiring antibiotics) could be distinguished from ICB-induced colitis (requiring high-dose steroids). However, an unblinded design allows for informative censoring [42], which may have occurred for some control-arm *BRAF*-mutant cases. Acknowledging the survival data one can definitely argue that the study treatments were inadequate for certain study subpopulations. One of the only two significant findings—the CRP level might inform on ICB responsiveness—was not a prespecified analysis in the study protocol. Finally, our statistical power assumption—median PFS twice as long for the experimental arm (Supplementary Methods)—was not met.

In conclusion, the first-line METIMMOX concept for MSS-CRC patients with abdominal metastases was negative with regard to the primary outcome for the intention-to-treat population, which echoes data from other randomised trials of ICB added to first-line chemotherapy in MSS-CRC.

### Supplementary information

Supplementary information

## Data Availability

Requests for raw or analysed data will be reviewed by the study team and responded to within 2-3 weeks. The data generated in this study are subject to patient confidentiality in accordance with the General Data Protection Regulation of the European Union, and the transfer of data or materials will require approval from the Data Privacy Officer at Akershus University Hospital and in some occasions from the Regional Committee for Medical and Health Research Ethics of South-East Norway. Any shared data will be de-identified. Requests can be made to the corresponding author.
