# Supplementary material for: First-line oxaliplatin-based chemotherapy and nivolumab for metastatic microsatellite-stable colorectal cancer—the randomised METIMMOX trial
Source: Br J Cancer. 2024 Apr 25;130(12):1921–8. doi: 10.1038/s41416-024-02696-6 (PMC11183214; doi:10.1038/s41416-024-02696-6)
Supplement: Supplementary file 1 — Supplementary information [file 41416_2024_2696_MOESM1_ESM.docx]

**SUPPLEMENTARY METHODS**

**Procedures**

Patients with previously untreated, unresectable infradiaphragmatic metastases from microsatellite-stable (MSS) colorectal cancer (CRC) were enrolled at five hospitals in Norway: the sponsor Akershus University Hospital (*n* = 39), Sørlandet Hospital (*n* = 19), St. Olav’s Hospital (*n* = 13), Oslo University Hospital (*n* = 5) and Haukeland University Hospital (*n* = 4). The patients were block-randomised into treatment arms with ratio 1:1 following a computer-based allocation sequence with regard to primary tumour sidedness (right or left/rectum) and *RAS/BRAF* mutational status (wildtype or any mutation). The masked randomisation was done by the investigators within an interactive web response system implemented in the Viedoc electronic case report form (Viedoc Technologies AB, Uppsala, Sweden). Randomisation listings were generated by an independent statistician at the Clinical Trial Unit Research Support Services, Oslo University Hospital, using STATA (StatCorp LLC, College Station, TX, USA).

The presence of commonly tested mismatch repair proteins (MLH1, MSH2, MSH6 and PMS2) in tumour specimens was confirmed by the immunohistochemistry protocol established from external quality assessment by the Nordic immunohistochemical Quality Control society (nordiqc.org), alternatively, the absence of tumour microsatellite instability markers was proven by Genomic Quality Assessment-verified PCR analysis, both performed by accredited molecular pathology laboratories. The MSS tumour status was for patients with radiologic complete response analysed at least two more times with complementary assays, which included determination of tumour mutational burden (TMB) by DNA sequencing, performed at the Akershus University Hospital National Unit for Precision Medicine.

For tumour sequencing, including TMB analysis, DNA/RNA was extracted from formalin-fixed paraffin-embedded primary tumour biopsies using the AllPrep DNA/RNA FFPE Mini Kit (Qiagen) or Mag-Bind FFPE DNA/RNA 96 Kit (Omega Bio-tek) or from fresh-frozen biopsies from metastatic lesions using the AllPrep DNA/RNA/miRNA Universal Kit (Qiagen). DNA and RNA concentrations were determined by a Qubit fluorometer (Thermo Fisher Scientific) and the quality was assessed by the Infinium FFPE QC Kit (Illumina) for DNA or the TapeStation HS RNA Kit (Agilent) for RNA. Sequencing was performed with the TruSight Oncology 500 DNA/RNA Assay (Illumina). The targeted exon coverage for DNA samples was 600-1850×. The DNA sequence data were analysed by the TruSight Oncology 500 Local App version 2.2 Software (Illumina). Further processing was done using the Personal Cancer Genome Reporter [1] followed by manual quality check to remove sequencing artefacts and germline variants. TMB was calculated using only coding, non-synonymous single-nucleotide variants and insertions/deletions with variant allele frequency ≥5%, in accordance with the national molecular diagnostics procedures [2]. The effective panel size in megabases was used as the denominator in the TMB calculations.

**Statistical analysis**

The statistical plan incorporated adjustments to enable, prior to the data analysis, the removal of 5% enrolled cases with non-MSS-CRC and up to 15% subjects lost before a valid progression-free survival (PFS) event had been reached, increasing the sample size to 50 patients in each arm. The protocol stated that any deviation from this plan would be described and justified in the clinical study report. First, the logistics of routine MSS testing had been established at all molecular pathology laboratories in the nation when the study was launched (May 2018), averting inadvertent inclusion of non-MSS cases. Next, during the course of the study, the sponsor became assured that the logistics of the study conduct at all study sites would secure a valid PFS event to be recorded for all intention-to-treat subjects. The accrual of patients was therefore concluded when 40 patients had been enrolled into each study arm with 1:1 randomisation after this had been found legitimate by the blinded independent monitoring committee, as recommended [3].

As the primary analysis, PFS times were presented by Kaplan-Meier curves and median PFS times were compared between the study arms by two-sided log-rank test. The prespecified efficacy analyses were done on the intention-to-treat sample, excluding ineligible patients who had been mistakenly randomised or did not receive any study intervention [3], by that corresponding to the safety population that consisted of patients who received at least one dose of study treatment. Because the first two therapy cycles were identical in the control and experimental study arms (halfway towards the first radiographic reassessment), the per-protocol population included all subjects who adhered to treatment until the first reassessment to enable objective comparison of the regimens.

To determine associations between PFS and relevant patient variables, stratified by study arm, prespecified Cox proportional hazards regression models were estimated. First, unadjusted models were estimated for each variable of interest. The models contained the variable, study arm dummy and the interaction between these two. Next, an adjusted model, including all variables, dummy for study arm and all possible two-way interactions, was estimated and reduced for excessive interactions by the Bayesian information criterion. A significant interaction would imply that an association between the variable and PFS differed between the study arms. The results are presented as hazard ratio and corresponding 95% confidence interval (CI) with *p*-values for each variable, except for variables constituting an interaction term which is presented as regression coefficient and standard error. The proportional hazards assumption was assessed by a global test and Schoenfeld residuals.

**SUPPLEMENTARY RESULTS**

All participants had C-reactive protein (CRP) <60 mg/L at study entry, as per protocol, but it had increased above 60 in five patients at start of therapy. The CRP measures for the intention-to-treat population declined over the initial treatment with Nordic FLOX (*p* = 0.034; Supplementary Fig. S6) from median 13.0 mg/L (minimum, 0.7; maximum, 112; *n* = 76) at start of the first FLOX cycle to median 8.0 mg/L (minimum, 0.6; maximum, 141; *n* = 73) at start of the second FLOX cycle and median 6.0 mg/L (minimum, 0.5; maximum, 60; *n* = 73) at start of the third therapy cycle. The implication with regard to PFS is depicted in Supplementary Fig. S7. Experimental-arm patients with CRP within the reference limit (<5.0 mg/L) when starting nivolumab as the third therapy cycle (*n* = 17) reached median PFS 15.8 months (95% CI, 7.8-23.7), in contrast to those still with CRP ≥5 mg/L (*n* = 19) who had median PFS 4.4 months (95% CI, 3.4-5.4; *p* < 0.001). No difference in PFS was seen for control-arm patients with CRP within or above the reference limit at start of the third FLOX cycle (*p* = 0.51). Of note, the experimental-arm patients with CRP <5 mg/L after the initial two FLOX cycles had longer PFS (*p* = 0.0082) than the corresponding control group (*n* = 9) with median PFS 12.2 months (95% CI, 2.6-21.8).

The neutrophil counts declined over the initial treatment and were, for the intention-to-treat population, median 4.6 ×10^9^/L (minimum, 1.5; maximum, 12.0; *n* = 76) at start of the first FLOX cycle, median 3.0 ×10^9^/L (minimum, 0.8; maximum, 11.3; *n* = 70) at start of the second FLOX cycle and median 2.7 ×10^9^/L (minimum, 1.4; maximum, 11.0; *n* = 69) at start of the third therapy cycle (*p* < 0.0001). Four patients were given granulocyte-colony stimulating factor because of early treatment-induced neutropenia—their subsequent neutrophil measurements were omitted from this analysis. Among the included experimental-arm subjects starting nivolumab, those with neutrophil counts below the median value for the entire cohort (*n* = 23) reached median PFS 15.0 months (95% CI, 11.9-18.1), in contrast to those with counts above (*n* = 12) who had median PFS 5.7 months (95% CI, 0.0-12.7; *p* = 0.029). However, no difference in PFS was seen between the control and experimental groups of patients with neutrophil counts below median at any of the initial study visits.

**SUPPLEMENTARY REFERENCES**

1. Nakken S, Fournous G, Vodák D, Aasheim LB, Myklebost O, Hovig E. Personal Cancer Genome Reporter: variant interpretation report for precision oncology. Bioinformatics 2018;34:1778-1780.

2. Taskén K, Russnes HEG, Aas E, Bjørge L, Blix ES, CONNECT Public-Private Partnership Consortium, et al. A national precision cancer medicine implementation initiative for Norway. Nat Med 2022;28:885-887.

3. Fergusson D, Aaron SD, Guyatt G, Hébert P. Post-randomisation exclusions: the intention to treat principle and excluding patients from analysis. BMJ 2002;325:652-654.


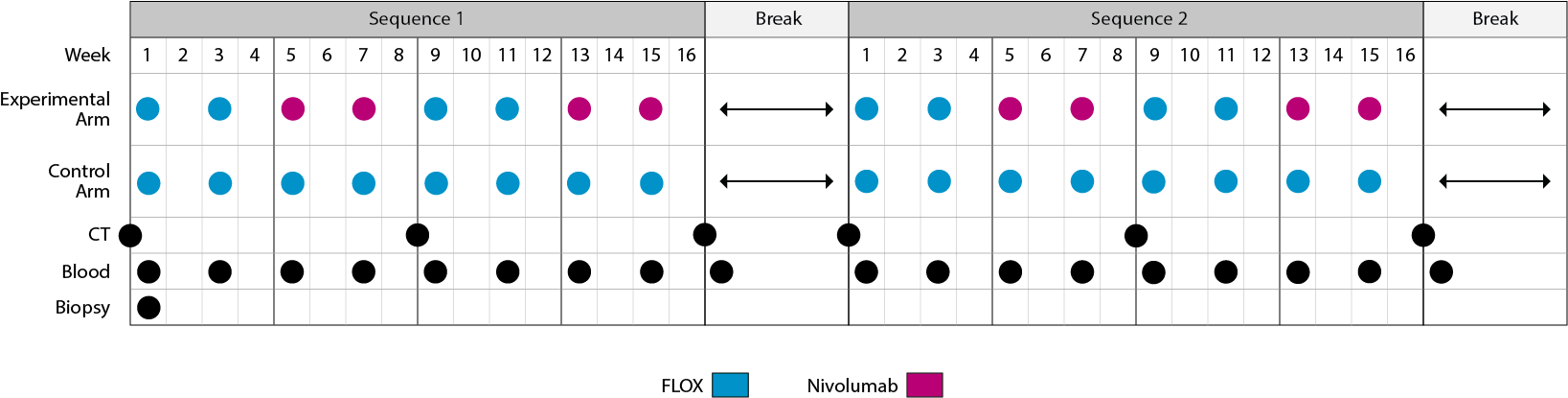


**Supplementary Fig. S1 The METIMMOX trial schedule.**

Circles indicate study visits with appendant activities. The treatment schedule consisted of intermittent periods of eight cycles. FLOX: oxaliplatin 85 mg/m^2^ day 1 and bolus 5-fluorouracil 500 mg/m^2^ and folinic acid 100 mg days 1-2; intravenous administration Q2W. Nivolumab: 240 mg flat dose; intravenous administration Q2W. During a break period, radiographic assessment (usually by computed tomography; CT), blood biobanking and visits were done every 8 weeks until disease progression and the treatment was reintroduced in a new sequence. Treatment sequences were continued until disease progression on ongoing therapy (progressive disease), an intolerable adverse event, consent withdrawal or death, whichever occurred first. Timing for blood and biopsy biobanking is indicated; investigations on the biobank material are not reported here.

**Supplementary Fig. S2 CONSORT flow diagram of the study cases.**

The trial enrolled 40 control-arm patients to FLOX and 40 experimental-arm patients to alternating two cycles each of FLOX and nivolumab. Four cases were screening failures or withdrew the informed consent before the first FLOX cycle was administered, leaving 76 intention-to-treat patients randomly allocated between the study arms. These 76 patients also constituted the safety population. Two control-arm *BRAF*-mutant cases had major protocol deviation following the initial two FLOX cycles, as the investigator chose another regimen from the third treatment cycle, at which time point the patients were censored. Five additional control-arm patients left the study before the first post-baseline radiographic assessment—one withdrew consent, one was found dead at home (autopsy was not done) and three had other intolerable events—resulting in 31 per-protocol cases. In the experimental arm, two patients left the study after the first FLOX cycle—one because of cardiac asystole (which was reversed) and the other from fatal colitis—resulting in 36 per-protocol cases.

|  |  | **Control arm**  **(*n* = 38)** | **Experimental arm**  **(*n* = 38)** |
| --- | --- | --- | --- |
| **Gene** | **Codon** | ***n*** | ***n*** |
| *RAS/BRAF* | Wildtype | 9 | 12 |
| *KRAS* | G12A | 1 | 2 |
|  | G12C | 2 | 2 |
|  | G12D | 1 | 3 |
|  | G12R | 0 | 1 |
|  | G12S | 1 | 2 |
|  | G12V | 3 | 6 |
|  | G13A | 1 | 0 |
|  | G13D | 1 | 4 |
|  | Q61H | 2 | 0 |
|  | Q61L | 1 | 0 |
|  | Q61R/L | 1 | 0 |
|  | K117X | 0 | 1 |
|  | A146P/T/V | 0 | 2 |
|  | A146X | 1 | 0 |
| *NRAS* | G13R/V | 2 | 0 |
|  | G13V | 1 | 0 |
|  | Q61R | 1 | 0 |
| *BRAF* | V600E/D | 10 | 3 |

**Supplementary Table S1.** The number of *RAS/BRAF* variants in the study arms.

|  | **Control arm** | | **Experimental arm** | | **Experimental arm**  ***versus* control arm** | |
| --- | --- | --- | --- | --- | --- | --- |
|  | **Hazard ratio**  **(95% CI)** | ***p*** | **Hazard ratio**  **(95% CI)** | ***p*** | **Hazard ratio**  **(95% CI)** | ***p*** |
| **Unadjusted model** | | | | | | |
| <60 years | 1 |  | 2.24  (0.72-7.01) | 0.17 | 2.24  (0.72-7.01) | 0.17 |
| ≥60 years | 1.88  (0.63- 5.59) | 0.25 | 1.04  (0.35-3.12) | 0.94 | 0.55  (0.28-1.09) | 0.087 |
| ≥60 *versus*  <60 years | 1.88  (0.44-8.11) | 0.40 | 0.46  (0.22-0.98) | 0.045 | 0.25  (0.07-0.93) | 0.039 |
| **Adjusted model** | | | | | | |
| <60 years | 1 |  | 4.09  (1.16-14.4) | 0.029 | 4.09  (1.16-14.4) | 0.029 |
| ≥60 years | 3.04  (0.82-11.2) | 0.096 | 2.11  (0.59-7.52) | 0.25 | 0.69  (0.33-1.45) | 0.33 |
| ≥60 *versus*  <60 years | 3.04  (0.61-15.1) | 0.17 | 0.52  (0.23-1.17) | 0.12 | 0.17  (0.04-0.76) | 0.021 |

**Supplementary Table S2.** Hazard ratios and 95% confidence intervals (CIs) derived from the interaction Study arm × Age.

The unadjusted models revealed that a progression-free survival (PFS) event was equally likely for patients younger and older than 60 years receiving chemotherapy only but significantly less likely (*p* = 0.045) for patients over 60 years of age receiving alternating short-course FLOX and nivolumab. Altogether, the older patient group had significantly lowered risk of progression when given the experimental study therapy (*p* = 0.039 for the interaction term). The adjusted model showed that the younger patient group experienced significantly better PFS when receiving the standard FLOX chemotherapy (*p* = 0.029). The model confirmed that patients from 60 years of age had significantly improved PFS when given alternating short-course FLOX and nivolumab (*p* = 0.021 for the interaction term).

**Supplementary Fig. S3 The non-significant interactions by patients variables.**

Hazard ratio (HR) and 95% confidence interval (CI) with regard to progression-free survival (PFS) and overall survival (OS).

*ECOG* Eastern Cooperative Oncology Group.

**
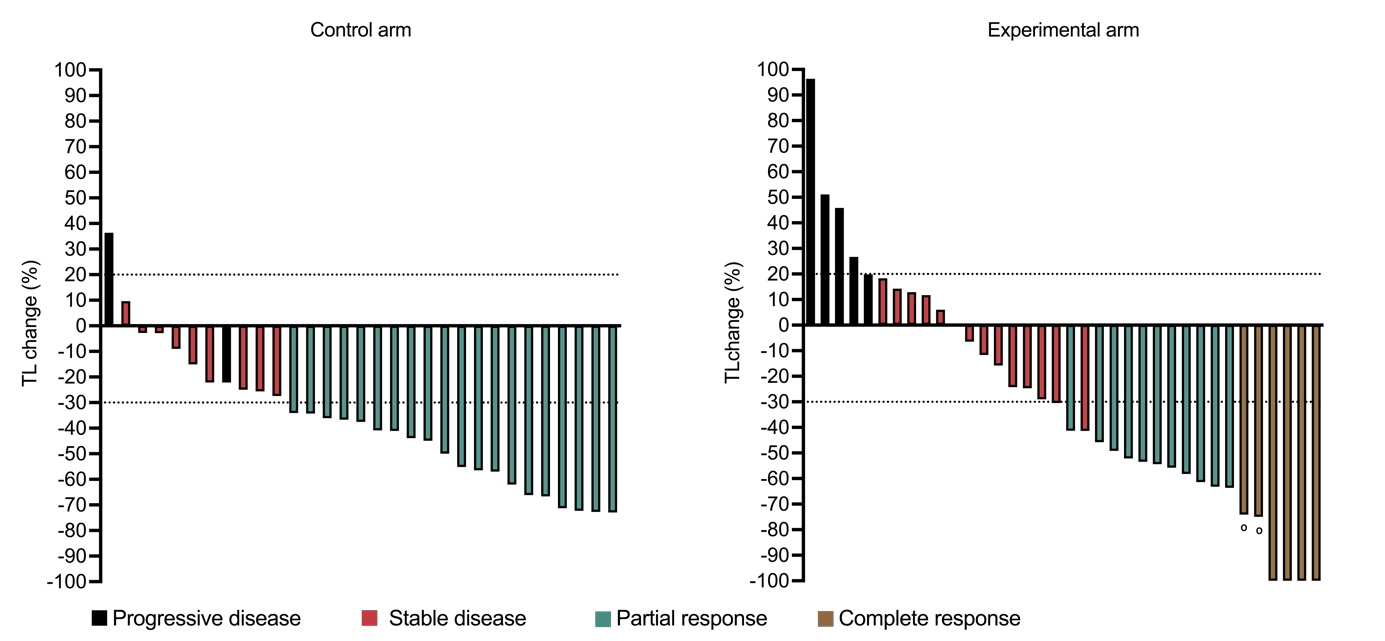
**

**Supplementary Fig. S4 Best overall responses in the per-protocol population of 67 cases.** Best percentage change in the sum of the longest target lesion (TL) diameters from baseline according to RECIST version 1.1. Circle: Patient had radiologic complete response of TL lymph node metastases.

|  |  | **CR+PR**  **(*n* = 37)** | **SD-PD**  **(*n* = 30)** | ***p*** |
| --- | --- | --- | --- | --- |
|  |  | ***n* (%)** | ***n* (%)** |  |
| Study arm | Control | 20 (54) | 11 (37) | 0.16 |
|  | Experimental | 17 (46) | 19 (63) |  |
| Age | <60 years | 9 (24) | 12 (40) | 0.17 |
|  | ≥60 years | 28 (76) | 18 (60) |  |
| Sex | Female | 16 (43) | 13 (43) | 0.99 |
|  | Male | 21 (57) | 17 (57) |  |
| ECOG performance status | 0 | 22 (59.5) | 16 (53) | 0.62 |
|  | 1 | 15 (40.5) | 14 (47) |  |
| Primary tumour sidedness | Right | 10 (27) | 8 (27) | 0.97 |
|  | Left or rectum | 27 (73) | 22 (73) |  |
| *RAS/BRAF* status | Wildtype | 12 (32) | 8 (27) | 0.61 |
|  | Mutant | 25 (68) | 22 (73) |  |
| Number of metastatic sites | 1-2 | 25 (68) | 14 (47) | 0.085 |
|  | >2 | 12 (32) | 16 (53) |  |
| Involved liver | No | 4 (11) | 4 (13) | 0.75 |
|  | Yes | 33 (89) | 26 (87) |  |

**Supplementary Table S3.** Comparison of best overall tumour responses by patient variables.

*CR* complete response, *ECOG* Eastern Cooperative Oncology Group, *PR* partial response, *SD* stable disease, *PD* progressive disease.

|  | **Control arm (*n* = 38)** | | **Experimental arm (*n* = 38)** | | | | |
| --- | --- | --- | --- | --- | --- | --- | --- |
|  | **FLOX** | | **FLOX** | | **Nivolumab** | | |
| **Cycles, median number**  **(minimum; maximum)** | 8 (1; 24) | | 6 (1; 20) | | 6 (0; 19) | | |
|  | **Grade 3** | **Grade 4** | **Grade 3** | **Grade 4** | **Grade 2^1^** | **Grade 3** | **Grade 4** |
|  | ***n* (%)** | ***n* (%)** | ***n* (%)** | ***n* (%)** | ***n* (%)** | ***n* (%)** | ***n* (%)** |
| **Patients with any event** | 25 (66) | 10 (26) | 21 (55) | 11 (29) | 2 (6)^1^ | 16 (42) | 0 |
| **Cardiovascular** |  |  |  |  |  |  |  |
| Arterial hypertension | 1 (3) | 0 | 0 | 0 | – | – | – |
| Atrial fibrillation | 0 | 0 | 1 (3) | 0 | – | – | – |
| Cardiac asystole | 0 | 0 | 0 | 1 (3) | – | – | – |
| Dehydration | 1 (3) | 0 | 1 (3) | 0 | – | – | – |
| Oedema | 0 | 0 | 1 (3) | 0 | – | – | – |
| **Gastrointestinal** |  |  |  |  |  |  |  |
| Diarrhoea | 1 (3) | 0 | 2 (5) | 0 | – | 1 (3) | 0 |
| Ileus | 2 (5) | 1 (3) | 1 (3) | 2 (5) | – | – | – |
| Nausea | 1 (3) | 0 | 1 (3) | 0 | – | – | – |
| **Haematologic** |  |  |  |  |  |  |  |
| Anaemia | 1 (3) | 0 | 1 (3) | 0 | – | – | – |
| Febrile neutropenia | 1 (3) | 0 | 5 (13) | 2 (6) | – | – | – |
| Neutropenia | 13 (34) | 9 (24) | 9 (24) | 6 (16) | – | – | – |
| Thrombocytopenia | 0 | 1 (3) | 1 (3) | 0 | – | – | – |
| Venous thromboembolism | 4 (11) | 0 | 2 (5) | 0 | – | 5 (13) | 0 |
| **Inflammatory** |  |  |  |  |  |  |  |
| Fever | 0 | 0 | 1 (3) | 0 | – | – | – |
| Infection | 4 (11) | 1 (3) | 5 (13) | 0 | – | – | – |
| **Metabolic** |  |  |  |  |  |  |  |
| Hypokalaemia | 0 | 0 | 1 (3) | 0 | – | – | – |
| **Neurologic** |  |  |  |  |  |  |  |
| Fatigue | 4 (11) | 0 | 3 (8) | 0 | – | – | – |
| Pain | 1 (3) | 0 | 1 (3) | 0 | – | – | – |
| Paraesthesia | 1 (3) | 0 | 0 | 0 | – | – | – |
| Spinal cord compression | 1 (3) | 0 | 0 | 0 | – | – | – |
| **Renal** |  |  |  |  |  |  |  |
| Renal failure | 1 (3) | 0 | 0 | 0 | – | 1 (3) | 0 |
| Urinary retention |  |  |  |  |  | 1 (3) |  |
| **Respiratory** |  |  |  |  |  |  |  |
| Dyspnoea | 1 (3) | 0 | 0 | 0 | – | – | – |
| **Administration** |  |  |  |  |  |  |  |
| Infusion-related reaction | 2 (5) | 0 | 0 | 0 | – | 0 | 0 |
| **Immune-mediated** |  |  |  |  |  |  |  |
| Arthritis | – | – | – | – | – | 1 (3) | 0 |
| Hyperglycaemia | – | – | – | – | – | 4 (11) | 0 |
| Hypophysitis | – | – | – | – | – | 1 (3) | 0 |
| Increased hepatic enzymes | – | – | – | – | 2 (6) | 2 (6) | 0 |
| Pneumonitis | – | – | – | – | – | 1 (3) | 0 |
| Rash | – | – | – | – | – | 3 (8) | 0 |

**Supplementary Table S4.** Adverse events in the intention-to-treat population.

^1^Grade 2 events were recorded only for immune-related hepatotoxicity (increased hepatic enzymes). In addition to the listed events, two grade 5 events were recorded following chemotherapy administration (nivolumab had not been given).


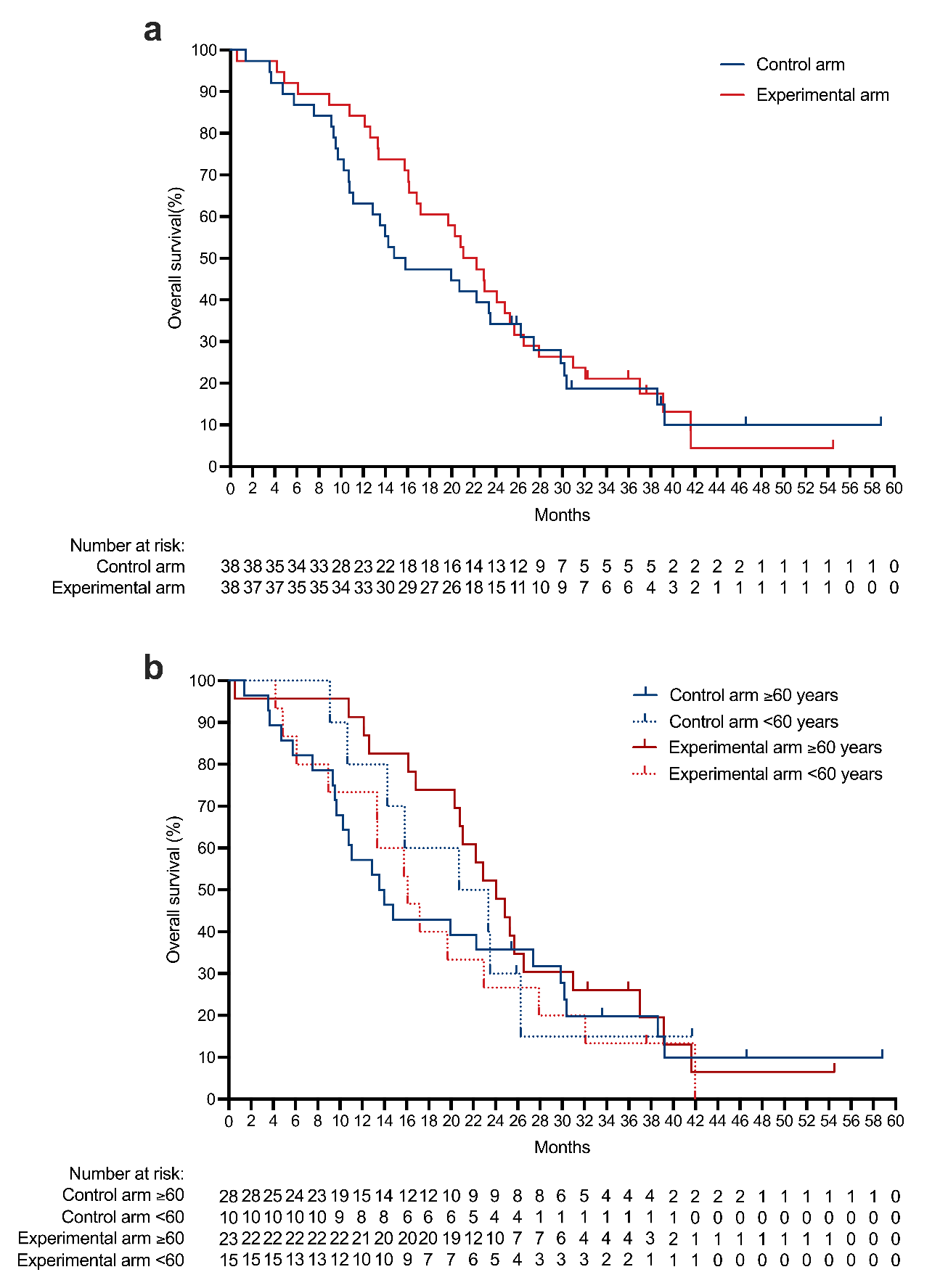


**Supplementary Fig. S5 Kaplan-Meier curves of overall survival for the intention-to-treat population.**


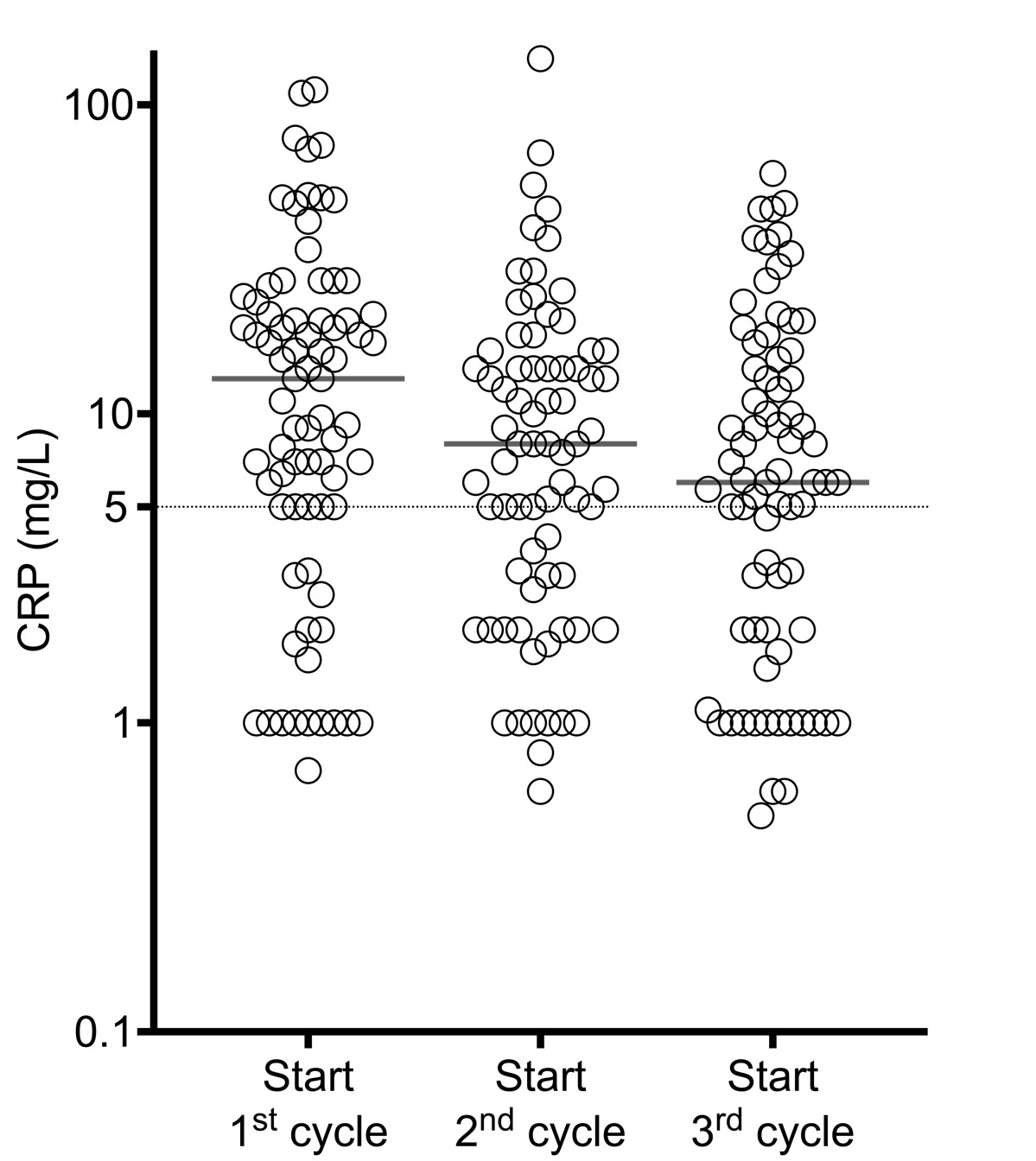


**Supplementary Fig. S6 C-reactive protein (CRP) for the intention-to-treat population.** Circulating levels at start of the first (*n* = 76), second (*n* = 73) and third (*n* = 73) therapy cycles.


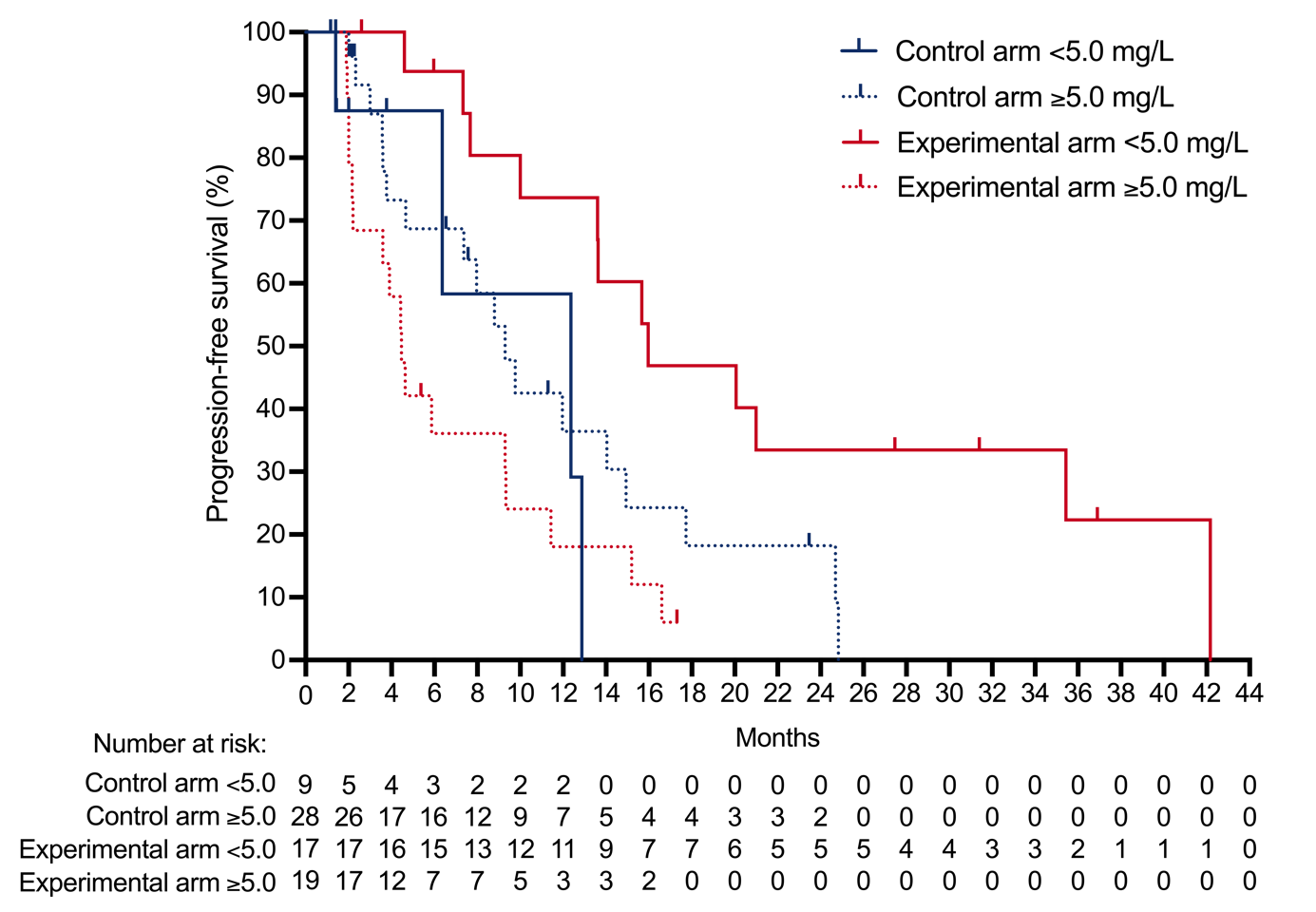


**Supplementary Fig. S7 Kaplan-Meier curves of progression-free survival for the intention-to-treat population at start of the third therapy cycle.** The 73 cases were stratified by study arm and the level of C-reactive protein; <5.0 mg/L: within the reference limit, ≥5 mg/L: above the reference limit.
